# Genetic Mapping and Identification of Candidate Genes for a New Multi-Branching Mutant *mbm1* in *Brassica napus*

**DOI:** 10.3390/ijms27062611

**Published:** 2026-03-12

**Authors:** Shiqin Li, Bao Li, Zhengfeng Zhang, Nanwei Chen, Xinmei Li, Mei Li, Tonghua Wang, Xiaoying Zhao

**Affiliations:** 1 Longping Agricultural College, Hunan University, Changsha 410082, China; lsq18711009479@163.com (S.L.); libao@hunaas.cn (B.L.); zzhengfeng8866@163.com (Z.Z.); nanweichen@hnu.edu.cn (N.C.); leexinmei@163.com (X.L.); limei1230@126.com (M.L.); 2 Hunan Province Key Laboratory of Plant Functional Genomics and Developmental Regulation, Hunan Engineering and Technology Research Center of Hybrid Rapeseed, College of Biology, Hunan University, Changsha 410082, China; 3 Crop Research Institute, Hunan Academy of Agricultural Sciences, Changsha 410125, China

**Keywords:** *Brassica napus*, branch number, BSA-seq, RNA-seq, KASP marker

## Abstract

Branch number is an important agronomic trait that determines the number of siliques per plant and yield in rapeseed (*Brassica napus*). However, the complex genetic and molecular mechanisms regulating the formation of branch number in rapeseed remain unclear. In a previous study, we isolated an EMS-induced multi-branching mutant (*mbm1*). Here, agronomic trait analysis showed that the *mbm1* mutant produced more effective primary branches, total silique number, and yield per plant compared with the wild type. Genetic analysis indicated that the multi-branching trait of the *mbm1* mutant is controlled by incompletely dominant gene(s) and follows nuclear inheritance. As a result of combined BSA-seq-based mapping and RNA-seq profiling, two candidate genes, B*naC03G0462000ZS* (*BnaC03.GAE6*) and *BnaC03G0491900ZS* (*BnaC03.MEE14*), were identified based on sequence variation and expression differences. KASP marker analysis confirmed that there are significant associations between these two gene loci and the multi-branching trait. Hybrid progeny derived from crosses between the *mbm1* mutant and commercial cultivars L329 and ZS11 showed significantly improved yield due to increased effective branch number. Together, these results provide important genetic resources and a theoretical foundation for further elucidating the genetic regulatory network of branching in rapeseed and for developing high-yielding rapeseed varieties with ideal plant architecture.

## 1. Introduction

Rapeseed (*Brassica napus*) is a globally important oil crop with substantial value for both human consumption and animal feed [1]. Yield per unit area in rapeseed is largely determined by individual plant productivity, which is directly influenced by three key components: thousand-seed weight, seeds per silique, and siliques per plant [2]. Among these, siliques per plant is often considered the most significant contributor to yield in most genetic backgrounds, though this cannot be generalized to all backgrounds. However, as a complex trait, it is highly susceptible to environmental variation and remains challenging to target directly for genetic improvement. In contrast, branch number represents a more stable trait across different environments, and it can effectively regulate siliques per plant, thereby affecting yield [3]. Various studies have indicated that there is a significant positive correlation between rapeseed yield and the number of effective branches [4]. In particular, the number of primary effective branches has been shown to exert the strongest effect on yield. Optimizing the structure and number of primary effective branches under varying planting densities can enhance silique number per plant and ultimately improve yield [5].

Given the important role played by the primary effective branch number in rapeseed yield formation, researchers have conducted extensive studies on this trait [6]. For instance, Shah et al. (2018) demonstrated that a mutation in the termination codon of the K domain of *Bna.AP1.A02* alters the expression of downstream target genes such as *Bna.TFL1* and *Bna.FUL* in the shoot apical meristem (SAM) of rapeseed, thereby significantly affecting plant architecture-related traits, including flower morphology, plant height, and branch number [7]. Zheng et al. identified two homologs of *MORE AXILLARY GROWTH 1* (*MAX1*), namely *BnaA03g22900D* and *BnaC03g26960D*, in the rapeseed genome using sequence alignment. Targeted mutagenesis of these genes via CRISPR/Cas9 led to a multi-branching phenotype in the resulting mutant plants [8]. The *Bna.TFL1* gene is present in five copies located on chromosomes A02, A10, C02, C03, and C09, corresponding to the loci *BnaA02G0014100ZS*, *BnaA10G0288700ZS*, *BnaC02G0013900ZS*, *BnaC03G0016500ZS*, and *BnaC09G0608000ZS*. Individual knockout of these five genes revealed that the branch number of the *BnaC09G0608000ZS* single mutant was not significantly different from that of the wild-type. In contrast, the other four single mutants all exhibited reduced branch numbers compared with the wild-type [9]. Overexpression of *FATTY ACID EXPORT 6* (*BnFAX6*) in transgenic plants promoted axillary bud development into lateral branches, raising the number of effective branches by about 109.8–114.8% [10]. There are five *BRANCHED1* (*BRC1*) genes in rapeseed, including *BnaA01.BRC1*, *BnaC01.BRC1*, *BnaA03.BRC1*, *BnaC03.BRC1*, and *BnaC05.BRC1*. These genes are predominantly expressed in leaf axils. Simultaneous knockout of *BnaA01.BRC1*, *BnaC01.BRC1*, *BnaA03.BRC1*, and *BnaC03.BRC1* leads to excessive branching [11]. Furthermore, knocking out all five *Bna.BRC1* genes produces a highly branched plant architecture in rapeseed [12]. Recently, the gene *SHOOT APICAL MERISTEM ENLARGER1* (*SAME1*) was cloned and shown to promote primary branch number and silique number on the main inflorescence. The elevated expression of *SAME1* represses the expression of *BnaC06.ARR15* and expands the expression domain of *BnaA09.WUS*, leading to an enlarged SAM and, consequently, increased branch and silique number on the main inflorescence [13]. Whether additional genes are involved in the regulation of rapeseed branch number beyond the known *TFL1*, *BRC1*, and *SAME1* pathways remains to be further explored.

We previously isolated a multi-branching mutant, *mbm1*, in rapeseed through ethyl methanesulfonate (EMS) mutagenesis [14]. In this study, we performed an integrated analysis of the agronomic traits and genetics of the *mbm1* mutant. Using bulked segregant analysis sequencing (BSA-seq), RNA sequencing (RNA-seq), and KASP marker assays, we identified *BnaC03G0491900ZS* (*BnaC03M.EE14*) and *BnaC03G0462000ZS* (*BnaC03.GAE6*) as the candidate genes underlying its phenotype. Analysis of the agronomic traits in F_1_ hybrid combinations of *mbm1* with commercial rapeseed cultivars L329 and ZS11 demonstrated the potential application of *mbm1* in rapeseed breeding. The results of this study constitute important genetic resources and theoretical foundations for breeding new high-yielding rapeseed hybrids with appropriate branching architecture.

## 2. Results

### 2.1. The mbm1 Mutant Exhibited Increased Branch Number, Total Silique Number, and Yield per Plant

**Figure 1:**
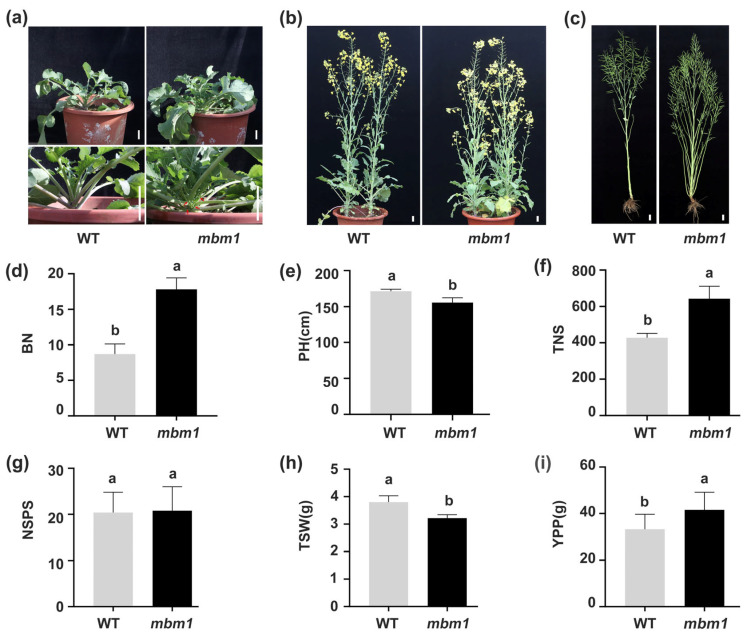
Phenotypic characteristics of the mutant *mbm1* and the wild-type (WT) plants. (**a**,**b**) Plant phenotypes at the seedling (**a**) and full-flowering (**b**) stages, the red arrow in (**a**) indicates the position of the basal axillary bud. (**c**) Phenotype of plants at the maturity stage. (**d**–**i**) Agronomic traits of *mbm1* and WT at the maturity stage, including branch number (BN) (**d**), plant height (PH) (**e**), total number of siliques (TNS) (**f**), number of seeds per silique (NSPS) (**g**), thousand-seed weight (TSW) (**h**), and yield per plant (YPP) (**i**). All values are presented as mean ± standard deviation (*n* = 10). Significant differences were determined by Student’s *t*-test. The unbracketed letters a and b are significantly different (*p* < 0.05). Scale bar = 5 cm.

The *mbm1* mutant was isolated from the polima cytoplasmic male sterility (CMS) maintainer line 2B following EMS mutagenesis [14]. Axillary buds and vegetative branches became visible in *mbm1* plants around the nine-leaf stage (Figure 1a). At both the full-flowering and maturity stages, *mbm1* developed significantly more branches than the wild-type parent 2B (WT) (Figure 1b,c). At the maturity stage, the number of primary effective branches in *mbm1* was 17.8 ± 1.62, more than double that of the WT (8.7 ± 1.42) (Figure 1d, Table S1). Analysis of other agronomic traits showed that the plant height of *mbm1* was significantly reduced compared with the WT (Figure 1e, Table S1). The mutant flowered slightly earlier and had a somewhat shorter growth period (Figure S1a,b). Silique length was also slightly shorter relative to the WT (Figure S1c,d, Table S1). Total silique number was significantly higher in *mbm1*, approximately 1.5 times that of the WT (Figure 1f, Table S1). No significant difference was observed in seeds per silique (Figure 1g, Table S1), whereas thousand-seed weight was lower in the mutant than in the WT (Figure 1h, Table S1). Ultimately, yield per plant was significantly increased in *mbm1*, at about 1.2 times that of the WT (Figure 1i, Table S1). Together, these results demonstrate that the *mbm1* mutant exhibits a marked increase in branch number and total silique number, resulting in higher yield per plant.

### 2.2. Genetic Characteristics of the Branch Number Trait in the mbm1 Mutant

**Figure 2:**
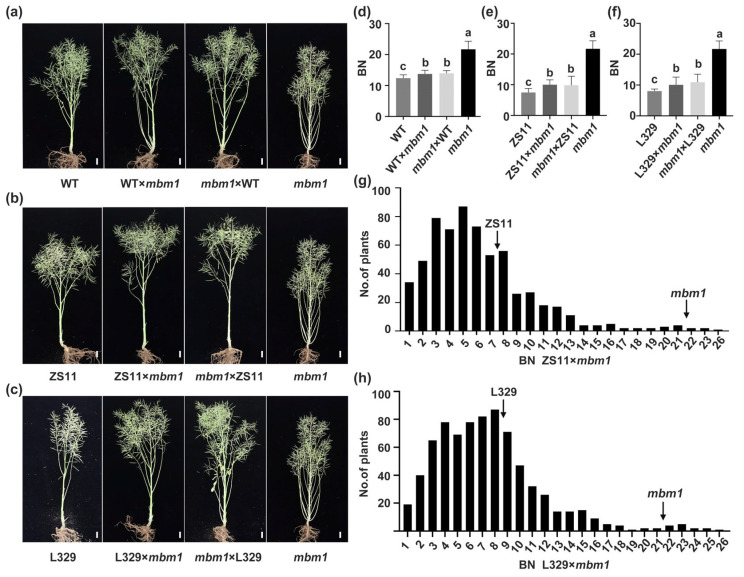
Branch phenotypes of the F_1_ hybrids and the F_2_ generations. (**a**–**c**) Phenotypes of reciprocal F_1_ hybrids between *mbm1* and WT (**a**), ZS11 (**b**), and L329 (**c**) at the maturity stage. (**d**–**f**) Branch number in reciprocal F_1_ hybrids between *mbm1* and WT (**d**), ZS11 (**e**), and L329 (**f**). Data are presented as mean ± SD (*n* = 18). Any two of the unbracketed letters a, b and c are significantly different (*p* < 0.05, Tukey’s test). (**g**,**h**) Frequency distribution of branch number in the F_2_ populations derived from crosses between *mbm1* and ZS11 ((**g**); 632 plants) and between *mbm1* and L329 ((**h**); 774 plants). Scale bar = 10 cm. BN: branch number.

To investigate the genetic basis of the increased branch number in the *mbm1* mutant, reciprocal crosses were performed between *mbm1* and wild-type (WT) plants, and branch number was assessed in the resulting F_1_ progeny. No significant difference in branching was observed between F_1_ plants from WT × *mbm1* and those from *mbm1* × WT crosses. However, the branch number in both F_1_ populations was significantly higher than that in the WT parent, yet it was lower than that in the *mbm1* mutant, exhibiting an intermediate phenotype (Figure 2a,d and Figure S2a). These results indicate that the multi-branching trait in *mbm1* is governed by incompletely dominant nuclear gene(s) and is not affected by cytoplasmic factors.

To further verify this genetic model, we performed reciprocal crosses between *mbm1* and the commercial cultivars ZS11 and L329. The resulting F_1_ hybrids—ZS11×*mbm1*, *mbm1*×ZS11, L329×*mbm1*, and *mbm1*×L329—were evaluated for branch number. Statistical analysis showed that the F_1_ plants from ZS11×*mbm1* and *mbm1*×ZS11 had approximately 10.00 and 9.83 branches, respectively, numbers that are significantly lower than those of *mbm1* (~21.72). Although a statistically significant difference in branch number was observed between the F_1_ progeny and ZS11, the branch numbers in the F_1_ progeny were biased toward that of ZS11 (~7.44) (Figure 2b,e, Figure S2b, Table S2). Similarly, the F_1_ progeny from reciprocal crosses between L329 and *mbm1* (L329×*mbm1* and *mbm1*×L329) produced significantly fewer branches than the *mbm1* parent, with averages of approximately 10.06 and 10.94 branches, respectively. Although a statistically significant difference in branch number was observed between the F_1_ progeny and L329, the F_1_ progeny exhibited a branch number phenotype similar to that of L329 (~8) (Figure 2c,f, Figure S2c, Table S2). Taken together, these results further support that the multi-branching trait in *mbm1* exhibits incomplete dominance and follows nuclear inheritance.

To determine whether the multi-branching trait in *mbm1* is quantitative or qualitative, we statistically analyzed the branching phenotypes in F_2_ segregating populations derived from F_1_ hybrids of ZS11×*mbm1* and L329×*mbm1*. In the ZS11×*mbm1* F_2_ population, the branch numbers from 632 individual plants showed a continuous distribution, with a small proportion exhibiting transgressive segregation phenotypes (Figure 2g). The Kolmogorov–Smirnov test for normality applied to the branching trait yielded a *p*-value of 0.000 (<0.05), indicating a significant deviation from normal distribution. Similarly, in the L329×*mbm1* F_2_ population, branch numbers from 774 individual plants also displayed a continuous distribution along with transgressive segregation in a subset of plants (Figure 2h). The branching trait in this population similarly deviated significantly from a normal distribution (*p* = 0.000). This non-normal distribution suggests that the trait fits a major gene plus polygene genetic model. These results demonstrate that the multi-branching trait of the *mbm1* mutant is a quantitative trait, and it follows a nuclear inheritance pattern.

### 2.3. Genetic Mapping of Multi-Branching Trait in mbm1 Using BSA-Seq

To map the genes associated with the multi-branching trait in *mbm1*, we performed BSA-seq analysis using an F_2_ population derived from F_1_ of L329×*mbm1*. From the F_2_ population, 50 high-branching plants (with 12 or more branches) and 50 low-branching plants (with 3–5 branches) were selected to form the high-branching number bulk (HB) and low-branching number bulk (LB), respectively. Sequencing was conducted on the HB and LB and the parental lines L329 and *mbm1*. After quality control filtering, a total of approximately 151.73 Gb of data was obtained, with HB, LB, L329, and *mbm1* contributing 53.90 Gb, 46.98 Gb, 24.63 Gb, and 26.22 Gb, respectively, corresponding to coverage depths of 53.9×, 46.98×, 24.63×, and 26.22× (Table S3). The average Q30 and Q20 values reached 98.10% and 93.95%, and the average GC content was 36.59% (Table S3). Through ensuring alignment with the ZS11 reference genome and using the GVCFtyper algorithm for joint variant calling across multiple samples, a total of 5,186,556 single nucleotide polymorphisms (SNPs) were identified.

**Figure 3:**
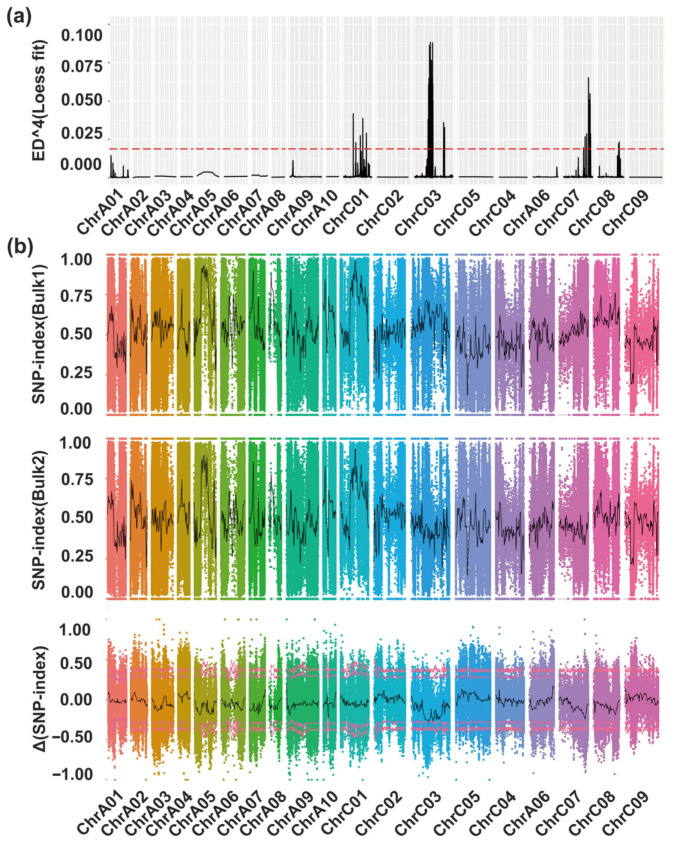
Mapping of the *mbm1* mutant using bulk segregant analysis coupled with resequencing. (**a**) Mapping interval identified by Euclidean distance (ED) analysis across chromosomes. The Y-axis represents the local weighted linear regression (LOESS)-fitted ED^4^ value, and the X-axis indicates the physical position along each chromosome. The red line corresponds to the threshold, calculated as 3 × (standard deviation of fitted values) + median of fitted values, where “fitted values” were derived from applying LOESS to the ED^4^ dataset. (**b**) Mapping interval based on Δ(SNP-index) distribution across chromosomes. The Y-axis shows Δ(SNP-index), and the X-axis denotes the physical position on chromosomes. The inner and outer red lines indicate the 95% and 99% confidence level thresholds, respectively.

Using the Euclidean Distance (ED) method to perform an association analysis of the target trait, SNPs with genotypic differences were utilized to calculate the read depth of each nucleotide in the HB and LB, and the ED value for each SNP was computed. The ED^4^ was then used as the association value to reduce background noise. Finally, local linear regression (LOESS) was applied to fit the ED^4^ values. Intervals where the ED value exceeded the threshold line (0.0187) were considered to be candidate regions associated with the target trait. The results showed that intervals exceeding the threshold were located on chromosomes C01, C03, C07, and C08, with the highest ED value and the longest interval observed on chromosome C03 (Figure 3a), suggesting that these candidate regions may be associated with the target trait. Based on this, further analysis was conducted on the intervals significantly associated with the target trait. On chromosome C01, five significantly associated intervals (referred to as ED-C01) were detected, with a total length of approximately 0.38 Mb. On chromosome C03, 30 significantly associated intervals (referred to as ED-C03) were detected, with a total length of approximately 7.53 Mb. On chromosome C07, 18 significantly associated intervals (referred to as ED-C07) were detected, with a total length of approximately 1.94 Mb. On chromosome C08, three significantly associated intervals (referred to as ED-C08) were detected, with a total length of approximately 0.14 Mb (Table S4).

Next, we conducted association analysis for the target trait using the Δ(SNP-index) method. Following filtering based on missing rate and sequencing depth, a total of 1,700,738 high-confidence SNP markers were obtained. Two significantly associated Δ(SNP-index) intervals were identified, located on chromosomes A10 and C03. The interval on chromosome C03 spanned approximately 0.60 Mb, while that on chromosome A10 covered about 0.20 Mb (Figure 3b). Comparison of the significant Δ(SNP-index) interval on chromosome C03 with the above-defined ED-C03 interval revealed that one ΔSNP-index interval fell entirely within the ED-C03 region. This interval, designated ES-C03, is located at 35,524,886–36,127,337 bp on chromosome C03 and has a length of 602,451 bp (Table S5). A total of 11 SNPs were screened (Table S6). In parallel, we screened SNPs from other ED significant association intervals outside ES-C03 that exhibited pronounced SNP-index differences (|ΔSNP-index| > 0.5, *p* < 0.05), as described previously [15,16]. We identified 5 SNPs from ED-C03 and 24 SNPs from ED-C07, while no SNPs were found in ED-C08 or ED-C01 (Table S7). In total, 40 candidate SNPs were selected from all target trait association intervals. These SNPs will be utilized for further identification of candidate genes underlying the multi-branching trait in *mbm1*.

### 2.4. Identification of Differentially Expressed Genes in Axillary Buds Between mbm1 and WT Using RNA-Seq

**Figure 4:**
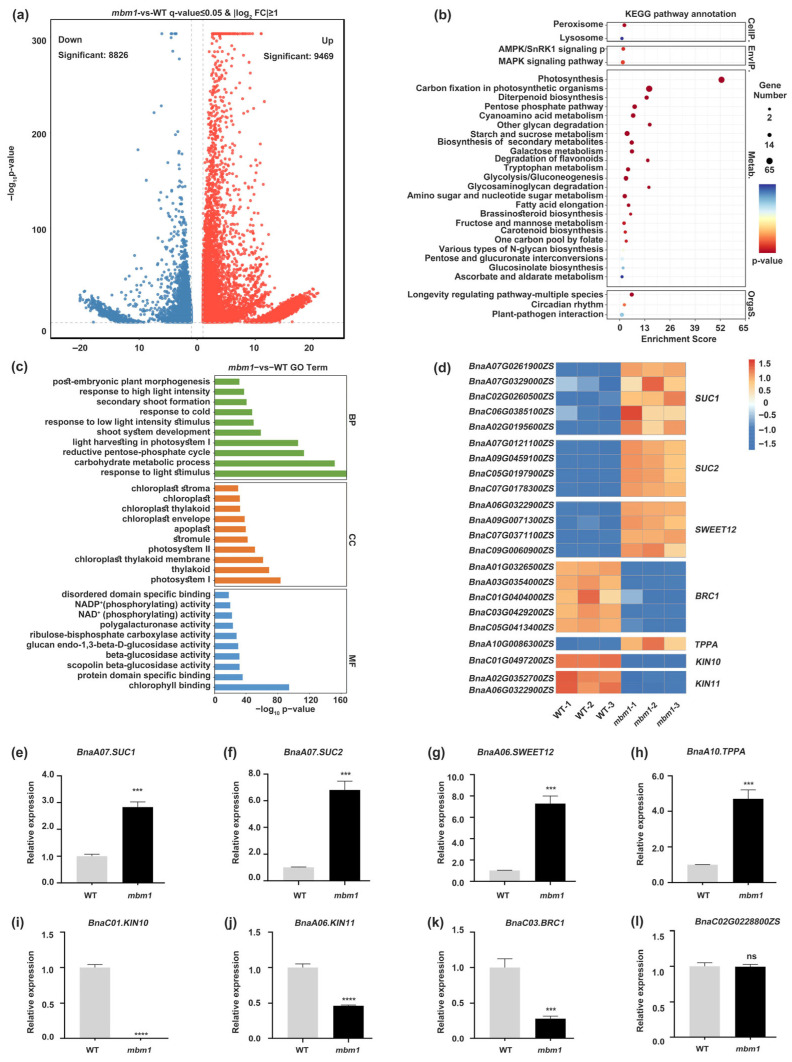
Differentially expressed genes in axillary buds in *mbm1* versus WT. (**a**) Volcano plot of all significantly differentially expressed genes (DEGs) in *mbm1* versus WT. DEGs were screened using the following criteria: upregulated genes with *q* ≤ 0.05 and log_2_FoldChange ≥ 1, and downregulated genes with *q* ≤ 0.05 and log_2_FoldChange ≤ −1. (**b**) KEGG pathway enrichment analysis displaying the top 30 significantly enriched terms for DEGs in *mbm1* versus WT. (**c**) GO functional enrichment analysis showing the top 30 significantly enriched terms for DEGs in *mbm1* versus WT. (**d**) Clustering heatmap of a subset of significant DEGs. Labels on the left correspond to rapeseed gene IDs, while labels on the right indicate their homologous genes in Arabidopsis. (**e**–**l**) mRNA levels of the indicated genes in axillary buds of *mbm1* and WT. The gene expression was normalized to *BnaACTIN7* expression. Bars represent the standard deviations of three independent experiments. Error bars indicate SD, *n* = 3. Statistically significant differences are shown: *** *p* < 0.001, **** *p* < 0.0001 (Student’s *t*-test). ns: no significant differences.

To further investigate the genetic and molecular mechanisms underlying the multi-branching phenotype of *mbm1*, we conducted RNA-seq analysis using axillary buds from *mbm1* and WT plants. A total of 18,295 significantly differentially expressed genes (DEGs) (*q* ≤ 0.05, |log_2_FoldChange| ≥ 1) were identified, with 9469 genes upregulated and 8826 downregulated in the *mbm1* mutant compared with the WT (Figure 4a, Tables S8 and S9). The KEGG pathway analysis revealed that the DEGs were predominantly associated with metabolic processes, particularly those related to carbohydrate and sugar metabolism, including carbon fixation in photosynthetic organisms (65 DEGs), starch and sucrose metabolism (31 DEGs), the pentose phosphate pathway (23 DEGs), glycolysis/gluconeogenesis (22 DEGs), and amino sugar and nucleotide sugar metabolism (19 DEGs) (Figure 4b, Table S10). The GO functional enrichment analysis further highlighted significant enrichment in terms such as response to light stimulus (262 DEGs), carbohydrate metabolic process (165 DEGs), the reductive pentose-phosphate cycle (69 DEGs), shoot system development (61 DEGs), secondary shoot formation (31 DEGs), post-embryonic plant morphogenesis (22 DEGs), chlorophyll binding (63 DEGs), beta-glucosidase activity (32 DEGs), and polygalacturonase activity (29 DEGs) (Figure 4c, Table S11). Taken together, the KEGG and GO enrichment analyses suggest that alterations in carbohydrate and sugar metabolism pathways may underpin the multi-branching trait in *mbm1*, providing a potential mechanistic basis for axillary bud outgrowth.

Sugars act as an energy source, and it is well-known that they promote bud outgrowth in many species [17]. Therefore, we focused on identifying differentially expressed genes associated with sugar metabolism. Our analysis revealed that the expression of five *SUCROSE-PROTON SYMPORTER 1* (*SUC1*) and four *SUC2* genes, which encode a key sucrose transporter [18], as well as four *SUGARS WILL EVENTUALLY BE EXPORTED TRANSPORTERS* (*SWEET12*) genes, encoding sucrose efflux transporters [19], and one *TREHALOSE-PHOSPHATE PHOSPHATASE A (TPPA)* gene, involved in trehalose-6-phosphate (Tre6P) metabolism [20], was significantly upregulated in *mbm1* (Figure 4d). Several studies have shown that inhibiting sucrose non-fermenting-related kinase (SnRK1) is essential to activating biosynthetic pathways in growing tissues in response to sucrose availability [21]. In line with this, we observed that the expression of *KIN10* and *KIN11*, encoding catalytic α-subunits of SnRK1 [22], was markedly downregulated in the *mbm1* mutant (Figure 4d). Furthermore, the transcript levels of five *BRC1* genes, which act as key negative regulators of branching [23], were also significantly reduced in the *mbm1* mutant (Figure 4d). To validate these findings, we selected eight genes to be the subjects of quantitative reverse-transcription PCR (qRT-PCR) analysis. Consistent with the RNA-seq data, the expression of *BnaA07G0261900ZS* (*BnaA07.SUC1*), *BnaA07G0121100ZS* (*BnaA07.SUC2*), *BnaA06G0322900ZS* (*BnaA06.SWEET12*), and *BnaA10G0086300ZS* (*BnaA10.TPPA*) was increased in the axillary buds of *mbm1* compared with the wild type (Figure 4e–h). In contrast, the expression of *BnaC01G0497200ZS (BnaC01.KIN10*), *BnaA06G0322900ZS* (*BnaA06.KIN11*), and *BnaC03G0429200ZS* (*BnaC03.BRC1*) was decreased (Figure 4i–k). No expression difference was observed for the negative control gene *BnaC02G0228800ZS* (Figure 4l). Taken together, these results indicate that the expression of genes related to sugar metabolism is altered in the *mbm1* mutant.

**Figure 5:**
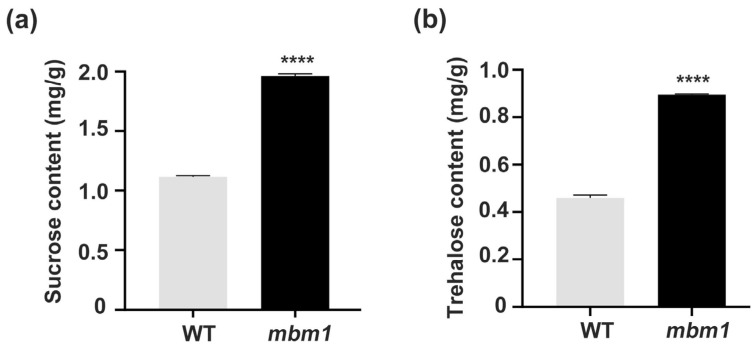
Sucrose and trehalose contents increased in the axillary buds of the *mbm1* mutant. Sucrose (**a**) and trehalose (**b**) contents in axillary buds of *mbm1* and WT plants. Both WT and *mbm1* mutant plants were cultivated in soil for two months. Axillary bud samples were then collected from individual plants, with three buds taken from each plant along the stem from the base to the upper region for measurement of sucrose and trehalose levels. Error bars indicate SD, *n* = 3. Statistically significant differences are shown: **** *p* < 0.0001 (Student’s *t*-test).

To determine whether the transcriptional changes in sugar-related genes led to altered sugar levels in *mbm1*, we quantified sucrose and trehalose in axillary buds. Both sugars were significantly elevated in *mbm1* compared with the wild type (Figure 5). Together, these results suggest that enhanced sugar accumulation in axillary buds likely contributes to the multi-branching phenotype of *mbm1*.

### 2.5. Association Analysis of BSA-seq and RNA-seq Data to Identify Candidate Genes Related to the Multi-Branching Trait in mbm1 Mutant

To further identify candidate genes associated with the multi-branching trait, we performed an integrative analysis combining the genes linked to the 40 SNPs identified through BSA-seq (Tables S6 and S7) and the DEGs from RNA-seq data (Tables S8 and S9). Among the genes associated with the 40 SNPs, 13 exhibited significant expression differences between *mbm1* and the wild type. Of these, two were located within the ED-C03 interval, one in the ES-C03 interval, and ten in the ED-C07 interval (Table S12). We then analyzed the expression levels of these 13 candidate genes in axillary buds. Four genes, namely *BnaC03G0501200ZS*, *BnaC07G0434100ZS*, *BnaC07G0437300ZS*, and *BnaC07G0451100ZS*, showed very low expression levels, with the Transcripts Per Million (TPM) values all being below 1 (Table S8). Therefore, these four candidate genes were excluded from further consideration. Subsequent in-depth analysis was focused on the remaining nine candidate genes: *BnaC07G0419600ZS*, *BnaC07G0436500ZS*, *BnaC07G0437400ZS*, *BnaC07G0409100ZS*, *BnaC07G0411200ZS*, *BnaC07G0419000ZS*, *BnaC07G0436900ZS*, *BnaC03G0462000ZS*, and *BnaC03G0491900ZS*.

Homologous gene functional annotation showed that *BnaC07G0419600ZS* has an Arabidopsis ortholog, *eIF4E1*, encoding eukaryotic translation initiation factor 4E1. This factor has been reported to regulate the nitrate signaling pathway and modulate nitrate uptake and metabolism [24]. *BnaC07G0436500ZS* corresponds to the cysteine-rich receptor-like kinase CRK29 in Arabidopsis, which, together with CRK28, is involved in mediating the immune responses associated with cell death [25,26]. The ortholog of *BnaC07G0437400ZS*, *AT4G21650*, belongs to the Subtilase (SBT) family and encodes a subtilisin-like serine protease; however, its specific function remains uncharacterized (https://yanglab.hzau.edu.cn/BnTIR (accessed on 14 July 2025)). *BnaC07G0409100ZS* is orthologous to Arabidopsis *INOSITOL TRANSPORTER 4* (*INT4*), encoding a high-affinity H^+^:myo-inositol symporter [27]. *BnaC07G0411200ZS* corresponds to *AT4G16650*, a potential gene for cell cycle regulation [28]. *BnaC07G0419000ZS* corresponds to *ALUMINUM-ACTIVATED MALATE TRANSPORTER 12* (*ALMT12*), an R-type anion channel that mediates stomatal closure [29]. *BnaC07G0436900ZS* is orthologous to *CHLORORESPIRATORY REDUCTION 9* (*CRR9*), which encodes a conserved chloroplast stromal protein [30]. Whether these seven genes are involved in the regulation of branching has not been reported.

*BnaC03G0462000ZS* is orthologous to Arabidopsis *UDP-D-GLUCURONATE 4-EPIMERASE 6* (*GAE6*), a key enzyme in pectin biosynthesis that converts UDP-glucuronic acid to UDP-galacturonic acid [31]. Pectin is a fundamental component of the primary cell wall, and its abundance directly influences wall integrity and mechanical behavior [32]. Loss of *GAE6* function has been shown to reduce pectin content, compromising cell wall integrity and leading to fragile leaves [33], whereas its upregulation enhances pectin precursor synthesis and increases pectin content [34]. The mechanical properties of the cell wall are modulated by the methylation and polymerization states of pectin, particularly of homogalacturonan (HG) [35]. HG can form nanofilaments that transition from crystalline to expanded conformations upon demethylation, a process that facilitates cell expansion by altering wall extensibility [36]. Changes in cell wall architecture are known to affect its permeability, which is critical for the intercellular transport of solutes such as sugars [37]. In our study, we observed significantly elevated levels of sucrose and trehalose in the axillary buds of the *mbm1* mutant (Figure 5), suggesting altered sugar accumulation or transport dynamics. Given the established role of GAE6 in pectin metabolism, the documented impact of pectin on cell wall permeability, and the known influence of sugar availability on axillary bud outgrowth, we propose that *BnaC03.GAE6* may affect sugar transport efficiency by modulating cell wall mechanical properties, thereby contributing to branch formation. While direct mechanistic evidence is yet to be elucidated, this hypothesis is grounded in a logical chain of supported connections from pectin biosynthesis to cell wall function and sugar transport.

*BnaC03G0491900ZS* is orthologous to *MATERNAL EFFECT EMBRYO ARREST 14* (*MEE14*), which is expressed in the female gametophyte and required for early embryo development [38,39]. *MEE14* has been shown to co-localize with dormancy-related miRNAs, suggesting that it plays a potential role in seed dormancy regulation [40]. Functional studies in pecan indicate its involvement in female gametophyte development [41]. More recently, *MEE14* was found to possess adenylate cyclase activity, enabling cAMP production and implicating it in embryo development and abiotic stress responses [42]. Notably, its upregulation in rapeseed roots under hypoxia conditions points to it playing a possible role in energy metabolism [43]. Based on its expression patterns under energy-related stress, we hypothesize that *BnaC03.MEE14* may contribute to branching regulation, potentially through modulating energy metabolism.

**Figure 6:**
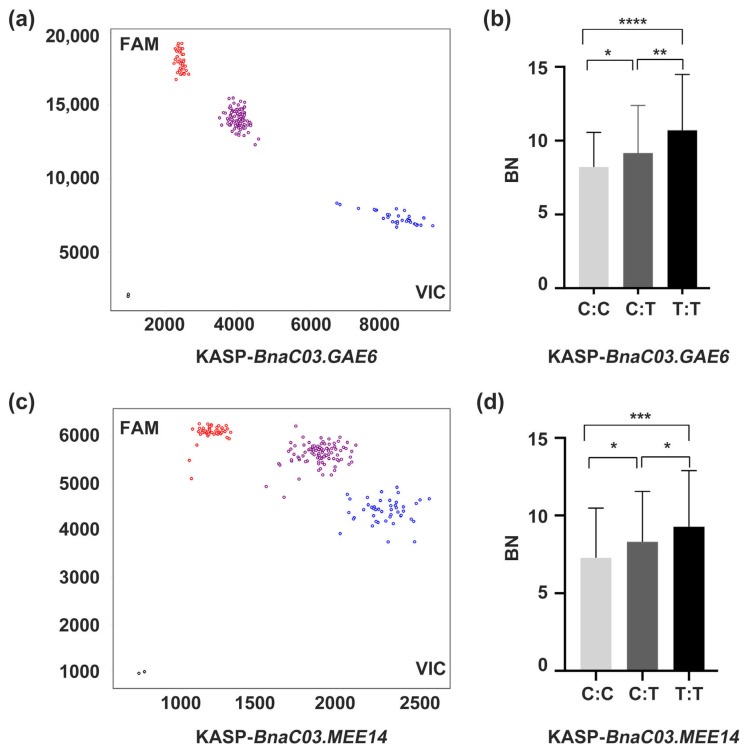
Genotyping of KASP markers KASP-*BnC03.GAE6* and KASP- *BnC03.MEE14* in the F_2_ population. (**a**) Scatter plot of genotyping for the KASP-*BnC03.GAE6* marker in the F_2_ population from L329 × *mbm1*. (**b**) Genotyping of the KASP-*BnC03.GAE6* marker and the corresponding branch numbers in the F_2_ population from L329 × *mbm1*. (**c**) Scatter plot of genotyping for the KASP-*BnC03.MEE14* marker in the F_2_ population from L329 × *mbm1*. (**d**) Genotyping of the KASP-*BnC03.MEE14* marker and the corresponding branch numbers in the F_2_ population from L329 × *mbm1*. Blue and red dots in (**a**,**c**) correspond to homozygous alleles, purple dots represent heterozygous alleles, and black dots indicate negative controls. Data in panels (**b**,**d**) are presented as mean ± standard deviation (SD). Significant differences were determined using Fisher’s LSD test (* *p* < 0.05, ** *p* < 0.01, *** *p* < 0.001, **** *p* < 0.0001).

Based on the functions or expression patterns of their Arabidopsis orthologs, we prioritized *BnaC03.GAE6* and *BnaC03.MEE14* for further validation. We developed kompetitive allele-specific PCR (KASP) markers for the candidate SNPs in these two genes (Table S13). Using the KASP-*BnaC03.GAE6* marker on 273 F_2_ plants derived from a cross between L329 and *mbm1*, we identified 69 wild-type (C:C), 142 heterozygous (C:T), and 62 mutant genotypes (T:T), with significant differences in branch number among genotypes (Figure 6a,b). Similarly, genotyping 317 F_2_ plants with the KASP-*BnaC03.MEE14* marker revealed 86 wild-type (C:C), 147 heterozygous (C:T), and 84 mutant plants (T:T), again showing significant variation in branch number (Figure 6c,d). These results indicate that these loci are significantly associated with the target trait and show cosegregation.

**Figure 7:**
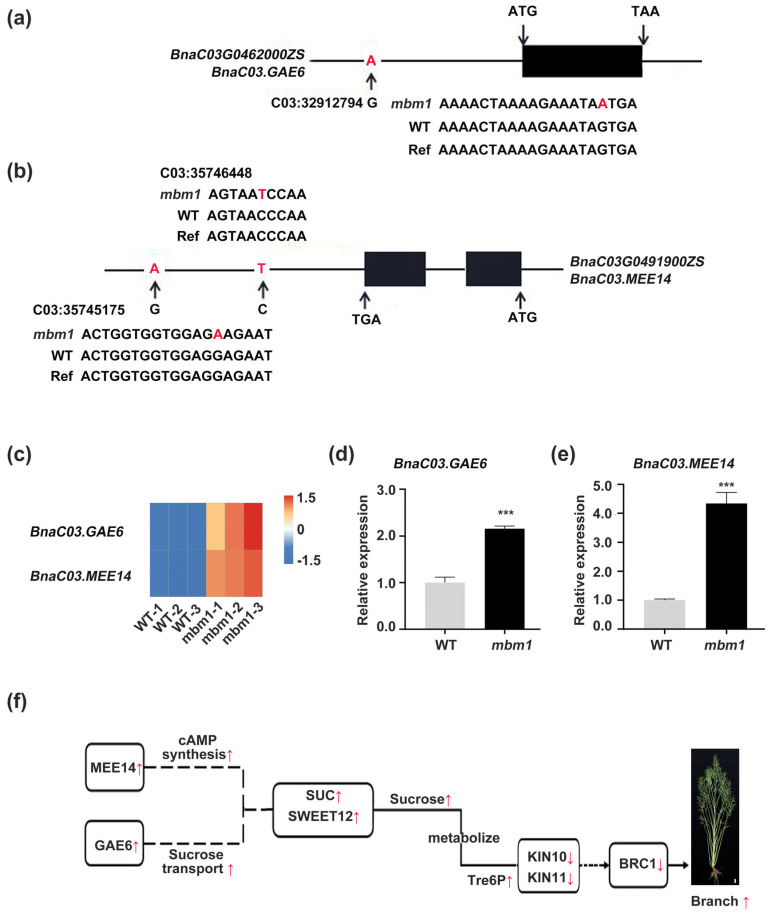
Gene structure and expression for candidate genes *BnaC03G0462000ZS* (*BnaC03.GAE6*) and *BnaC03G0462000ZS* (*BnaC03.MEE14*) associated with the multi-branching trait in *mbm1*. (**a**,**b**) Gene structures and mutation sites of *BnaC03G0462000ZS* (*BnC03.GAE6*) (**a**) and *BnaC03G0462000ZS* (*BnC03.MEE14*).The red letters indicate the mutated bases. (**b**). Exons and introns are represented by black boxes and lines, respectively. (**c**) Heatmap showing the expression levels of *BnC03.GAE6* and *BnC03.MEE14* in axillary buds of *mbm1* and WT. (**d**,**e**) qRT-PCR analysis of expression levels for *BnC03.GAE6* (**d**) and *BnC03.MEE14* (**e**) in axillary buds of *mbm1* and WT. The gene expression was normalized to *BnaACTIN7* expression. Bars represent the standard deviations of three independent experiments. Error bars indicate SD, *n* = 3. Statistically significant differences are shown: *** *p* < 0.001 (Student’s *t*-test). (**f**) Proposed regulatory model for multi-branching trait in the *mbm1* mutant. Dash lines indicate indirect regulation, solid lines represent direct regulation, red arrows indicate an increase or decrease, and boxes show rapeseed homologs of Arabidopsis genes: *MEE14* (*MATERNAL EFFECT EMBRYO ARREST 14*), *GAE6* (*UDP-D-GLUCURONATE 4-EPIMERASE 6*), *SUC* (*SUCROSE-PROTON SYMPORTER 1* and *2*), *SWEET12* (*SUGARS WILL EVENTUALLY BE EXPORTED TRANSPORTERS*), and *BRC1* (*BRANCHED1*).

The SNP in *BnaC03.GAE6* is a G-to-A mutation located 2907 bp upstream of the transcription start site (Figure 7a; Table S14), while that in *BnaC03.MEE14* is a C-to-T mutation 1327 bp downstream of the transcription end site (Figure 7b; Table S14). RNA-seq analysis showed that both genes were upregulated in *mbm1* axillary buds compared with the wild type (Figure 7c; Table S8), which was confirmed by qRT-PCR (Figure 7d,e). Taken together, these results suggest that *BnaC03.MEE14* and *BnaC03.GAE6* are candidate genes underlying the multi-branching phenotype of the *mbm1* mutant.

### 2.6. Potential Application of the Multi-Branching Mutant mbm1 in Breeding

The *mbm1* mutant displays superior agronomic performance, characterized by increased branch number, higher total silique number, and enhanced yield per plant (Figure 1; Table S1). To assess its breeding potential, we statistically analyzed agronomic traits in the F_1_ generation derived from crosses between *mbm1* and the cultivars ZS11 and L329 (Figure 2e,f, Figures S3 and S4; Table S2). In terms of total silique number, the F_1_ generation, although lower than that of *mbm1*, was significantly higher than that of ZS11 and L329 (Figures S3c and S4c; Table S2). Regarding seeds per silique, the F_1_ from ZS11 × *mbm1* was comparable to ZS11 and superior to *mbm1*, while the F_1_ from L329 × *mbm1* significantly exceeded the better parent, demonstrating obvious heterosis (Figures S3d and S4d; Table S2). For thousand-seed weight, the F_1_ generations of both ZS11 × *mbm1* and L329 × *mbm1* surpassed both parents, indicating a positive improvement trend (Figures S3e and S4e; Table S2). In terms of yield per plant, F_1_ yield was significantly higher than that of the commercial parents (Figures S3f and S4f; Table S2). These results demonstrate that *mbm1* can stably transmit its high-yield traits to hybrids, enabling F_1_ generation to achieve yield levels comparable to the high-yielding parent while substantially improving the performance of lower-yielding parents. This underscores *mbm1’s* promising potential for use in breeding applications.

In addition, the agronomic trait analysis results revealed that there were no significant differences in key yield-related traits—including branch number, total silique number, seeds per silique, thousand-seed weight, and yield per plant—between reciprocal crosses of *mbm1* with either ZS11 or L329 (Figure 2e,f, Figures S3 and S4; Table S2). These findings suggest that, in the context of heterosis breeding with the aim of improving plant architecture using these specific combinations, parental selection may not need to account for reciprocal cross effects. This provides greater flexibility in utilizing plant-type-related heterosis, at least for the combinations evaluated in this study.

## 3. Discussion

Branch number is a direct determinant of silique number per plant and ultimately yield in rapeseed. Previous studies have shown that the primary effective branch number contributes most significantly to yield, and modifying branch architecture can markedly increase siliques per plant [5]. Consistent with these findings, branch number indirectly promotes yield by increasing the effective silique number per plant, particularly under conventional planting densities [44]. In this study, the *mbm1* mutant exhibited a branch number that was 2.04 times that of the WT, along with an approximately 50% increase in total siliques per plant and a 25% rise in yield per plant (Figure 1), further confirming the positive role played by branch number in improving yield. Moreover, no significant differences in branching traits were observed between the reciprocal F_1_ hybrids of *mbm1* and the WT or the commercial varieties ZS11 and L329, indicating that the inheritance of branching traits is not affected by cytoplasmic effects (Figure 2a–f). This simplifies parental selection and facilitates the efficient utilization of this plant-type trait in hybrid breeding systems. Notably, transgressive heterosis for silique number on branches has been reported in F_1_ hybrids between compact- and loose-branching types [45], highlighting considerable potential for improving branch-related traits. In line with this, when *mbm1* was crossed with parents of different genetic backgrounds (ZS11, L329), its multi-branching and high-silique-number traits were stably inherited and effectively increased both branch number and total silique number in low-yielding parents (Figure 2b,c,e,f, Figures S3 and S4; Table S2). Furthermore, for traits such as seeds per silique and thousand-seed weight, the F_1_ hybrids generally exhibited transgressive heterosis or positive improvements. Consequently, the yield per plant of the F_1_ generation was significantly higher than that of ordinary parents such as ZS11 and L329, reaching a level comparable to the high-yielding *mbm1* itself (Figures S3 and S4; Table S2). These results demonstrate the potential applications of *mbm1* for crop improvement and breeding programs.

BSA-seq provides an efficient gene mapping approach that avoids genotyping every individual by pooling and sequencing subjects with extreme phenotypes, making it especially useful for mapping complex traits [46,47]. In this study, we integrated BSA-seq with RNA-seq to map the multi-branching mutant *mbm1*. Using the HB and LB and whole-genome sequencing data, we detected several intervals that were significantly associated with the multi-branching phenotype, with the most prominent located on chromosome C03 (Figure 3). From these regions, 40 candidate SNPs were selected (Tables S6 and S7). The candidate genes harboring these SNPs were then cross-referenced with DEGs from the RNA-seq data (*mbm1* vs. WT). This integrated analysis yielded nine candidate genes that were highly expressed in axillary buds and showed significant expression differences between *mbm1* and the WT. Based on the functional annotation of Arabidopsis orthologs and genotype–phenotype association analysis via KASP markers (Figure 6), two genes within the candidate interval on chromosome C03—*BnaC03.GAE6* and *BnaC03.MEE14*—were preliminarily identified as candidate genes associated with the multi-branching trait in *mbm1* (Table S14).

RNA-seq and qRT-PCR analyses revealed a significant upregulation of *BnaC03GAE6* and *BnaC03MEE14* expression in the *mbm1* mutant (Figure 7c–e). A key question remains as to how the SNPs in these genes affect their expression. Using PlantTFDB (https://planttfdb.gao-lab.org/ (accessed on 4 August 2025)), we examined cis-regulatory elements in and around the mutated genomic regions of *BnaC03GAE6* and *BnaC03MEE14*. The results showed that the G-to-A mutation located 2907 bp upstream of the *BnaC03GAE6* transcriptional start site falls within a transcription factor binding site (TFBS) for an AP2 family TF (Figure 7a, Table S15), suggesting that this mutation may interfere with TF binding and consequently affect *BnaC03GAE6* expression. In contrast, no TFBS was identified at the C-to-T mutation site 1327 bp downstream of the *BnaC03MEE14* transcriptional end site. A recent study reported that a natural mutation located 1.4 kb downstream of the coding sequence of the *SAME1* gene significantly enhanced its expression in the shoot apical meristem, leading to increased numbers of effective primary branches and siliques on the main inflorescence [13]. Based on this, we hypothesize that the downstream mutation in *BnaC03MEE14* may affect its expression by modifying local chromatin architecture or generating a novel regulatory element. Notably, a second G-to-A mutation exists 1273 bp downstream of this site, which lies within an ERF family TFBS (Figure 7b, Table S16). Previous studies have shown that plant enhancer-like modules can function through chromatin looping [48], and that genomic sequence variations—including point mutations and transposon insertions—can alter local chromatin accessibility and reshape gene regulatory networks [49]. We hypothesize that these two mutations near *BnaC03MEE14* may act synergistically: the upstream mutation could promote chromatin opening, while the downstream mutation alters ERF transcription factor binding. Together, they could form a reinforced enhancer-like module that more effectively engages with the *BnaC03MEE14* promoter via three-dimensional genomic interactions such as chromatin looping, ultimately leading to pronounced transcriptional upregulation. Nevertheless, the precise regulatory mechanisms underlying this upregulation require further validation through biochemical assays.

The other key question is how *BnaC03.GAE6* and *BnaC03.MEE14* regulate branching. *BnaC03.GAE6* is orthologous to Arabidopsis *GAE6*, which encodes a key enzyme in the biosynthesis of UDP-galacturonic acid, a primary precursor for pectin [31]. Altering the expression of *GAE6* is known to modulate pectin content, thereby influencing the mechanical properties and permeability of the cell wall [33,34,35,36]. Given that cell wall permeability is a critical determinant of intercellular sugar transport [37], and that sugars act both as essential energy sources and as signals that promote bud outgrowth across plant species [17], we hypothesized that changes in *BnaC03.GAE6* expression could impact branching by altering sugar levels in buds. Consistent with this hypothesis, we observed a significant upregulation in the expression of *BnaC03.GAE6* (Figure 7c,d) alongside significantly elevated levels of sucrose and trehalose (Figure 5) in the axillary buds of the *mbm1* mutant. Based on this correlative evidence and the well-established functional roles of its orthologs, we propose that *BnaC03.GAE6* contributes to the increased branching phenotype of *mbm1* by modulating pectin biosynthesis. This, in turn, is predicted to remodel the cell wall and subsequently influence intercellular sugar transport and accumulation in the bud. Future work involving direct measurements of cell wall composition and sugar flux will be necessary to experimentally validate this proposed mechanism. In addition, *BnaC03.MEE14* is an ortholog of *Arabidopsis MEE14*, a gene known to be required for female gametophyte and early embryo development [38,39]. Recent studies have shown that *MEE14* exhibits adenylate cyclase activity, produces cAMP, and plays a role in energy metabolism and stress responses [42,43]. In the present study, we observed that *BnaC03.MEE14* is upregulated in the axillary buds of the *mbm1* mutant (Figure 7c,e). Based on these findings, we hypothesize that *BnaC03.MEE14* may contribute to altered branching in *mbm1* by modulating energy metabolism and sugar accumulation, although further experiments are needed to establish a direct causal relationship.

Sugars promote bud outgrowth, in part, by downregulating *BRC1* expression [17]. BRC1 is a key repressor of axillary bud growth [23]. Tre6P, a phosphorylated disaccharide, serves as an intracellular signal of sucrose availability, integrating metabolic status with growth and developmental programs [50,51,52]. The heterologous expression of trehalose-6-phosphate phosphatase (TPP) in axillary buds reduces Tre6P levels and inhibits branching [17,53,54]. Elevated Tre6P levels downregulate *BRC1* expression, thereby promoting branching. Moreover, sucrose non-fermenting-related kinase 1 (SnRK1) has also been implicated in the transcriptional regulation of *BRC1* [55]. SnRK1 forms a heterotrimeric complex composed of a catalytic α subunit (KIN10/SnRK1α1 or KIN11/SnRK1α2) along with regulatory β and βγ subunits [22]. It is well-established that Tre6P inhibits SnRK1 activity [50,56,57,58,59]. Tre6P binds directly to KIN10, reducing the affinity of the activating kinase GRIK1 (Geminivirus Rep-Interacting Kinase 1) for KIN10. This results in decreased phosphorylation and activation of KIN10, ultimately lowering SnRK1 activity [59,60]. Multiple lines of evidence indicate that Tre6P-mediated inhibition of SnRK1 is required to activate biosynthetic processes in growing tissues in response to sucrose availability [21]. For example, sucrose-induced hypocotyl elongation is impaired in *Tre6P synthase 1* (*TPS1*) mutants and in plants overexpressing *SnRK1α1*, supporting the model that Tre6P-dependent suppression of SnRK1 permits cell elongation [61]. Additionally, sucrose-dependent entrainment of the circadian clock—which regulates processes such as hypocotyl growth—may also depend on Tre6P-mediated inhibition of SnRK1 [62]. In this study, we observed that sucrose metabolism-related genes, including *SUC1*, *SUC2*, and *SWEET12*, were upregulated in the axillary buds of the *mbm1* mutant (Figure 4d–g). Consistent with this, the levels of sucrose and trehalose were elevated in axillary buds (Figure 5). As expected, the expression of *KIN10*, *KIN11*, and *BRC1* was reduced in *mbm1* mutant buds compared with the wild type (Figure 4i–k). We therefore conclude that increased sucrose accumulation likely contributes at least partially to the multi-branching phenotype of the *mbm1* mutant.

Based on our results and those of previous studies, we propose a molecular regulatory network underlying the multi-branching trait in the *mbm1* mutant. This model involves two complementary pathways (Figure 7f): On the one hand, the upregulation of *BnaC03MEE14*, a gene encoding a protein with adenylate cyclase activity that catalyzes cAMP synthesis [42], may indirectly participate in sugar metabolism and induce the upregulation of *SUC*/*SWEET12*; this enhances sucrose transport capacity near axillary buds, thereby increasing intracellular sucrose levels and promoting Tre6P accumulation within the buds. On the other hand, upregulation of *BnaC03.GAE6*, which promotes pectin synthesis [31], could optimize cell wall structure or permeability. This improvement may facilitate sucrose unloading from the phloem or enhance intercellular transport [37], leading to an elevated concentration of local sucrose and subsequent Tre6P accumulation. Elevated levels of sucrose and Tre6P, in turn, suppress the expression of *KIN10* and *KIN11*, which lowers the expression of *BRC1* and ultimately promotes branch formation [50,56,57,58,59]. Interestingly, *TPPA*, which encodes a Tre6P-metabolizing enzyme [20], exhibits upregulated expression in the *mbm1* mutant (Figure 4h). Tre6P is a well-established signal metabolite that reflects cellular sugar status, and its hydrolysis via TPPA is known to contribute to sugar homeostasis [52]. Thus, the observed upregulation may hint at a feedback response to elevated sugar signaling. Nevertheless, further research is needed to validate the functions of the candidate genes *BnaC03.GAE6* and *BnaC03MEE14* and to fully elucidate the molecular regulatory network controlling branch development in rapeseed.

In conclusion, this study identified two candidate loci, *BnaC03.GAE6* and *BnaC03.MEE14*, underlying the multi-branching trait in the *mbm1* mutant by integrating BSA-seq mapping, RNA-seq differential expression analysis, and validation with KASP molecular markers. The evaluation of agronomic performance in F_1_ crosses of *mbm1* with the commercial cultivars ZS11 and L329 highlights its promising application in breeding programs. These findings offer valuable genetic resources and a theoretical basis for further deciphering the regulatory network underlying branching in rapeseed and for developing high-yielding varieties with optimized plant architecture.

## 4. Materials and Methods

### 4.1. Plant Materials and Growth Conditions

The multi-branching mutant *mbm1* was obtained from ethyl methanesulfonate (EMS) mutagenesis of seeds from the polima cytoplasmic male sterility maintainer line 2B [14]. The mutant was crossed with its wild-type parent 2B (WT) as well as with the commercial rapeseed cultivars L329 (Xiangyou 15) [63] and ZS11 [64]. The resulting progeny were self-pollinated to produce F_2_ populations.

Plants were cultivated in experimental fields located in Changsha City, Hunan Province, China (28.12° N, 112.59°E) and in greenhouses at Hunan University (28.17° N, 112.95° E). In the field trials, plants were arranged in rows with 30 cm between rows and 20 cm between plants within a row, with 10 plants per row. In the greenhouse, plants were grown in resin pots measuring 40 cm in diameter and 27 cm in height. All plants were grown under natural climate with natural light exposure. Rapeseed (*Brassica napus*) was used in this study. The polima cytoplasmic male sterility maintainer line 2B was kindly provided by Prof. Wusheng Peng (Yuan Longping High-tech Agriculture Co., Ltd., Changsha, China). The commercial rapeseed cultivars L329 (Xiangyou 15) and ZS11 were kindly provided by Prof. Zhongsong Liu (Hunan Agricultural University, Changsha, China).

### 4.2. Agronomic Traits Analysis

Agronomic traits, including plant height (PH), branch number (BN), total number of silique (TNS), silique length (SL), number of seeds per silique (NSPS), thousand-seed weight (TSW), and yield per plant (YPP), were measured and statistically analyzed following previously described methods [65]. Plants of *mbm1*, WT, ZS11, L329, and the different F_1_ hybrid combinations were harvested and air-dried naturally to determine thousand-seed weight and yield per plant. Days to flowering (DTF) and the whole growth period (WGP) were observed and recorded as described [66]. More than 10 plants of each material were used for statistical analysis. Statistical significance was assessed using GraphPad Prism 8.0.1 software.

### 4.3. Bulk Construction

For bulk construction, we evaluated the phenotypic distribution in branch number within an F_2_ population derived from a cross between *mbm1* and L329. Based on the observed distribution, two groups representing phenotypic extremes were selected: 50 plants with high branch numbers (12 or more branches) and 50 plants with low branch numbers (3–5 branches). These thresholds were determined according to the natural distribution of the trait in the population. Equal amounts of leaf tissue from each individual within each group were pooled into a single tube to generate two bulked pools: the high branch number bulk (HB) and the low branch number bulk (LB).

### 4.4. BSA-seq Analysis

BSA-seq analysis was conducted by HuaZhi Biotech Co., Ltd. (Changsha, China). HB and LB samples were ground into a fine powder using steel beads to ensure complete homogenization. DNA extraction was carried out using a magnetic bead-based method with a plant genomic DNA extraction kit (HuaZhi Biotech Co., Ltd., Changsha, China), which included 30 mL of Lysis Buffer LB, 60 µL of RNase, 1.2 mL of magnetic beads, 20 mL of Wash Buffer W1, 25 mL of Wash Buffer W2, and 20 mL of Elution Buffer TE.

The extraction procedure was as follows: first, 600 µL of preheated (65 °C) Lysis Buffer LB and 5 µL of RNase were added to the sample, and the tube was immediately inverted to mix. The mixture was incubated at 65 °C for 30 min, with inversion every 10 min to ensure thorough mixing. After cooling to room temperature, the sample was centrifuged at 12,000 rpm (13,400× *g*) for 10 min. Then, 400 µL of the supernatant was carefully transferred to a new centrifuge tube, followed by the addition of 300 µL of isopropanol and 20 µL of magnetic beads. The tube was inverted and mixed for 5 min, then subjected to magnetic separation for 2 min, after which the supernatant was discarded. Next, 750 µL of Wash Buffer W1 (with anhydrous ethanol added before use) was added, mixed by inversion for 1 min, and magnetically separated for 2 min before removing the supernatant. This washing step was repeated using 750 µL of Wash Buffer W2. After a brief centrifugation to collect residual liquid, the supernatant was discarded, and the tube was left open for 5 min to allow complete evaporation of ethanol. For DNA elution, 50–100 µL of Elution Buffer TE was added. The magnetic beads were thoroughly resuspended and incubated at 56 °C for 10 min, with intermittent mixing during incubation to improve elution efficiency. Finally, magnetic separation was performed for 2 min, and the supernatant containing the eluted DNA was transferred to a new 1.5 mL centrifuge tube for subsequent quality control.

DNA concentration was measured with a Qubit fluorometer (Invitrogen Qubit 4) (Thermo Fisher, Waltham, MA, USA), and integrity was assessed by 1% agarose gel electrophoresis. Samples meeting quality control standards (qualified DNA sample criteria: concentration ≥ 20 ng/μL, total amount ≥ 1 μg, volume 20–40 μL, or a clear main band with slight degradation and no impurities) were used to construct small-fragment libraries (300–500 bp). The DNA was fragmented with dsDNA Fragmentase, followed by end repair and 3’ end A-tailing. Fragmentation efficiency was verified by 2% agarose gel electrophoresis, and samples showing a distinct 300–500 bp band were carried forward. Sequencing adapters were ligated to the fragmented DNA using ligase, and ligation products were purified with magnetic beads. The purified product concentration was measured with the Qubit fluorometer; qualified samples were then subjected to PCR amplification. PCR products were size-selected using magnetic beads, and their concentration was again measured by Qubit. Fragment size distribution was confirmed by 2% agarose gel electrophoresis and further verified using a Qsep400 bio-analyzer (Houze Biotechnology, Hangzhou, China). Linear libraries were denatured into single strands and circularized. Linear DNA molecules that failed to circularize were digested, yielding single-stranded circular DNA libraries. The concentration of the circularized libraries was measured with the Qubit fluorometer, and qualified samples were processed further. Single-stranded circular DNA was amplified via rolling circle replication to form DNA nanoballs (DNBs), each containing over 300 copies. DNBs were loaded onto sequencing chips using the MGIDL-T7 loader (DNBSEQ-T7) (MGI Tech Co., Ltd., Shenzhen, China). Sequencing was performed using Joint Probe Anchor Polymerization technology.

Raw image data files from high-throughput sequencing were base-called to generate sequencing reads. Low-quality reads (where >50% of bases had quality score Q ≤ 20), adapter sequences, reads containing >5 ‘N’ bases, and reads shorter than 100 bp were removed by Fastp (v0.23.2). The remaining reads were aligned to the ZS11.V0 reference genome using Sentieon’s DNAseq pipeline (v202308.03), followed by sorting and duplicate marking. Variant calling was performed for each sample to generate gVCF files. A joint analysis of all gVCF files was conducted to obtain variant results for each individual in the population using the algorithm “GVCFtyper” in Sentieon. To ensure SNP accuracy, preliminary hard filtering was applied to the SNP sites from the joint analysis using the following criteria by GATK4: “QD < 2.0 || FS > 60.0 || MQ < 40.0 || SOR > 3.0 || MQRankSum < −12.5 || ReadPosRankSum < −8.0” [67]. Candidate loci were identified using the Euclidean Distance (ED) method [68] and the Δ(SNP-index) method [69]. The SNP-index was calculated using the “QTLseqr” package with a window size of 1 Mb. Threshold lines at the 95% and 99% confidence levels were set, and windows above the 95% confidence level were considered candidate intervals. SNPs were screened according to the following criteria: (1) using ZS11 as the reference genome, SNPs were those between mutant *mbm1* and wild-type and were homozygous; (2) SNPs involved C/G to T/A substitutions; (3) SNPs included important mutation types such as mutations in upstream and downstream regulatory regions, loss of stop codon, gain of stop codon, and non-synonymous mutations.

### 4.5. KASP Markers Analysis

KASP markers were developed based on polymorphic SNPs associated with the multi-branching phenotype. The KASP marker analysis was conducted by HuaZhi Biotech Co., Ltd. (Changsha, China). SNP genotyping was performed using KASP technology from Laboratory of the Government Chemist Ltd. (LGC, Teddington, United Kingdom). Three primers were designed: two allele-specific forward primers with fluorescent adapters and one common reverse primer. The fluorescent adapters used were FAM (5′-GAAGGTGACCAAGTTCATGCT-3′) and HEX/VIC (5′-GAAGGTCGGAGTCAACGGATT-3′). The assay also included two fluorescent probes and two corresponding quencher probes. The fluorescent probes were complementary to the quencher probes, and their sequences were identical to the adapter regions of the allele-specific forward primers.

KASP assays were performed on a Douglas ArrayTape genotyping platform. Each PCR reaction had a total volume of 0.8 µL, containing: 20–50 ng of dried sample DNA, 0.0013 µL each of the 100 µM allele-specific primers, 0.0033 µL of the 100 µM common primer, 0.3945 µL of 2× KASP Master Mix, and 0.3996 µL of ultrapure water. Amplification was performed in a water-bath thermal cycler (LGC, Teddington, United Kingdom) using a touchdown PCR protocol as follows: initial denaturation at 94 °C for 15 min; first amplification stage—10 cycles of 94 °C for 20 s and a combined annealing/extension step starting at 65 °C (decreasing by 0.8 °C per cycle) for 60 s, ending at 57 °C; second amplification stage—30 cycles of 94 °C for 20 s and 57 °C for 60 s. Following amplification, fluorescence of the KASP reaction products was detected using the ArrayTape scanning system. The fluorescence data were automatically processed and converted into genotype plots. The sequences of the primers are listed in Table S13, and the composition of the assay reaction system is detailed in Table S17.

### 4.6. RNA-seq Analysis

Two months after sowing in soil, at the nine-leaf stage, axillary buds from both the WT and the mutant *mbm1* were harvested and immediately frozen in liquid nitrogen. For each genotype, samples were collected from individual plants, with three axillary buds taken from each plant, spanning from the base to the upper stem. The plants used for collecting axillary buds are shown in Figure S5. The transcriptome sequencing and analysis were performed by HuaZhi Biotech Co., Ltd. (Changsha, China), with three biological replicates per genotype.

Clean reads from each sample were aligned to the ZS11 reference genome using HISAT2 (v2.2.1) [70]. Transcript abundance was estimated as Fragments Per Kilobase of transcript per Million mapped reads (FPKM) using RSEM software (v1.3.3) [71]. Differential gene expression analysis was performed with DESeq2. Genes were considered differentially expressed if they met the following thresholds: q-value < 0.05 and |log_2_FoldChange| ≥ 1, with fold changes calculated from log_2_-transformed expression values.

### 4.7. qRT-PCR Analysis

Total RNA was extracted using an RNA extraction kit (Accurate Biology, Changsha, China). cDNA was then synthesized by reverse transcription with the PrimeScript RT reagent Kit with gDNA Eraser (Takara Bio, Kyoto, Japan). Quantitative reverse transcription-PCR (qRT-PCR) was performed on a BioRad CFX96 Touch real-time PCR system with the SYBR Green Premix Pro Taq HS qPCR kit (Accurate Biology, Changsha, China), in accordance with the manufacturer’s instructions. *BnaACTIN7* served as the internal reference gene. The primer sequences used are listed in Table S18. All experiments were conducted with three biological replicates.

### 4.8. Sucrose and Trehalose Content Measurement

WT and *mbm1* mutant plants were grown in soil for two months. Axillary bud samples were then collected from individual plants, with three buds taken per plant along the stem from the base to the upper region for measuring sucrose and trehalose levels. Plants used for collecting axillary buds are shown in Figure S5.

For sucrose measurement, approximately 0.1 g of tissue was homogenized at room temperature in 0.5 mL of 80% ethanol extraction buffer. Following thorough grinding, the homogenate was promptly transferred to a centrifuge tube and incubated in an 80 °C water bath for 10 min, with intermittent shaking every 2–3 min. After cooling to room temperature, the mixture was centrifuged at 4000× *g* for 10 min at 25 °C, and the supernatant was collected. Sucrose was then extracted using the Plant Tissue Sucrose Content Assay Kit (Shanghai Sinobestbio Technology Co., Ltd., Shanghai, China) following the manufacturer’s protocol. The absorbance at 480 nm was recorded with a microplate reader (PerkinElmer). Sucrose concentration was quantified based on a sucrose standard. Three biological replicates were performed.

For trehalose measurement, about 0.1 g of tissue was homogenized at room temperature and combined with 1 mL of extraction buffer containing approximately 9.1% trichloroacetic acid (TCA). The mixture was incubated at room temperature for 45 min with intermittent shaking 3 to 5 times during the incubation period. After cooling, the sample was centrifuged at 8000× *g* at room temperature, and the supernatant was collected. Trehalose was then extracted using the Plant Tissue Trehalose Content Assay Kit (Shanghai Sinobestbio Technology Co., Ltd.) according to the supplied instructions. Absorbance was measured at 620 nm using a microplate reader (PerkinElmer), and trehalose content was determined by reference to a trehalose standard. Three biological replicates were conducted.

## Data Availability

The bulked segregant analysis sequencing (BSA-seq) and RNA sequencing (RNA-seq) data are available at http://bigd.big.ac.cn/ with the project number of PRJCA055317, and the accession number is CRA036898.

## Supplementary Materials

The following supporting information can be downloaded at: https://www.mdpi.com/article/10.3390/ijms27062611/s1.
