# Supplementary material for: Genetic Mapping and Identification of Candidate Genes for a New Multi-Branching Mutant mbm1 in Brassica napus
Source: Int J Mol Sci. 2026 Mar 12;27(6):2611. doi: 10.3390/ijms27062611 (PMC13027094; doi:10.3390/ijms27062611)
Supplement: Supplementary file 1 [file ijms-27-02611-s001.zip › LSQ et al-Supplemental figures S1-S5-final.pdf]

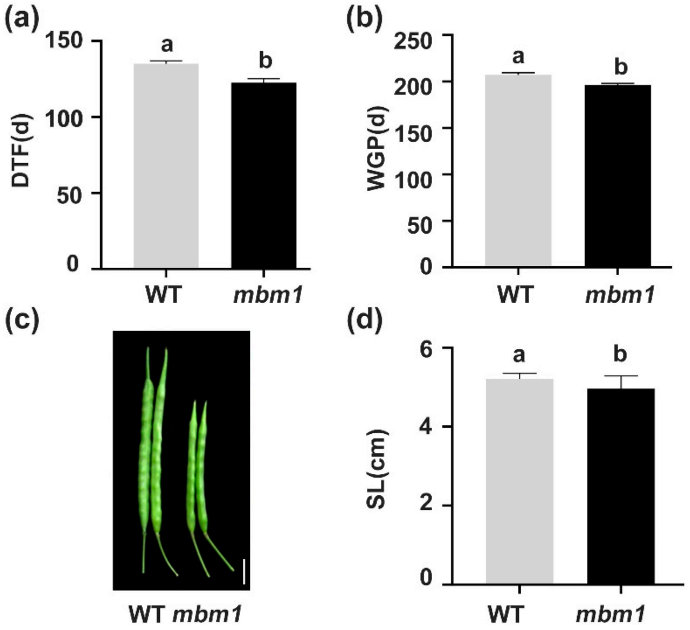

Figure S1. Phenotypic characteristics of mutant *mbm1* and wild-type (WT).

(a) Days to flowering (DTF). **(b)** Whole growth period (WGP). **(c)** Silique phenotypes at the maturity stage. **(d)** Silique length (SL) at the maturity stage. Data are presented as mean ± SD (n = 10). Significant differences were determined by Student's t-test. The unbracketed letters a and b are significantly different ($P < 0.05$). Scale bar = 1 cm.

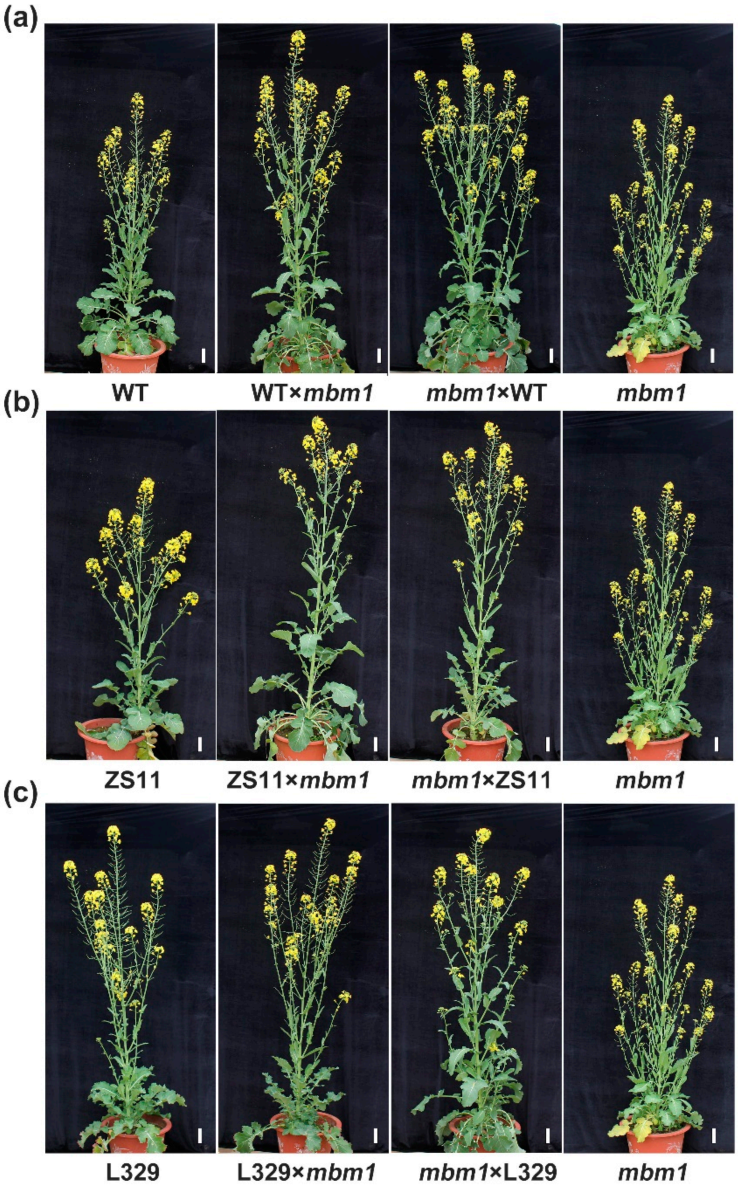

Figure S2. Phenotypes of F_1 hybrids between *mbm1* and WT, ZS11, and L329 at the full-flowering stage.

Phenotypes of reciprocal cross F_1 hybrids between *mbm1* and WT **(a),** ZS11**(b)**, and L329 **(c)** at the full-flowering stage. Scale bar = 10 cm

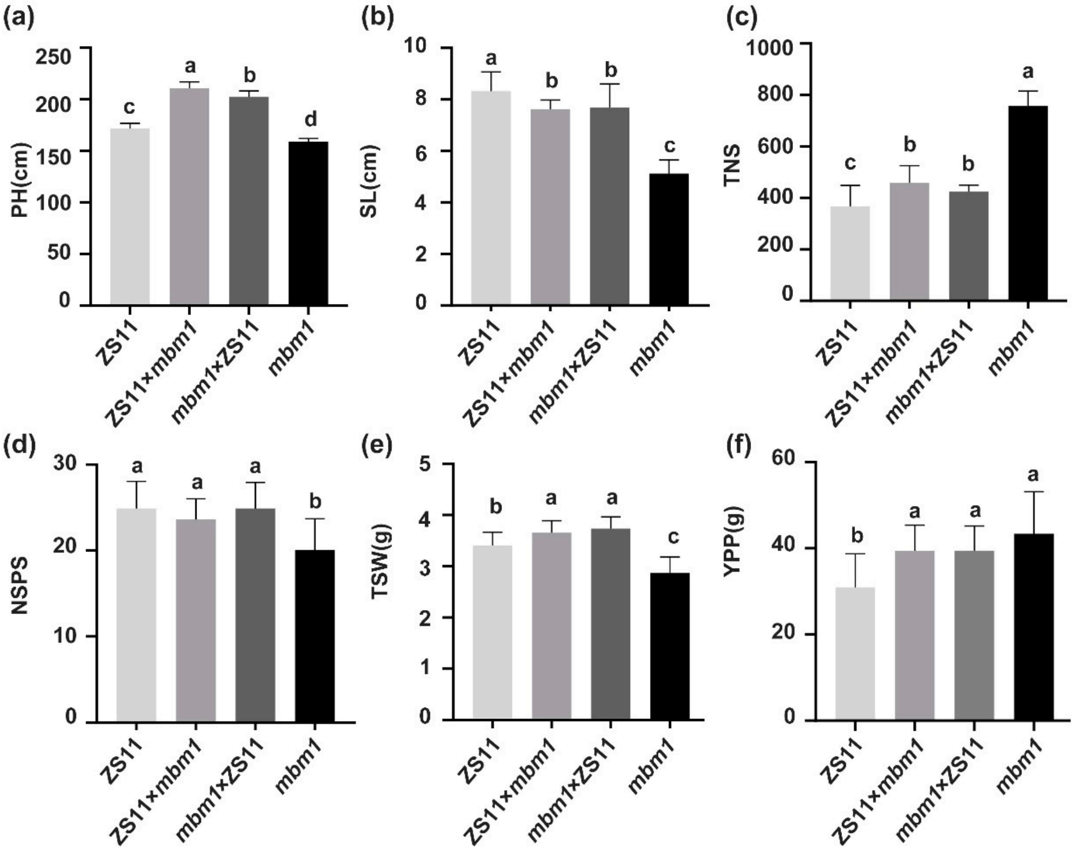

Figure S3. Agronomic traits of reciprocal cross F_1 hybrids between *mbm1* and ZS11.

(a) Plant height (PH). **(b)** Silique length (SL). **(c)** Total number of siliques (TNS). **(d)** Number of seeds per siliques (NSPS). **(e)** Thousand-seed weight (TSW). **(f)** Yield per plant (YPP). Data are presented as mean ± standard deviation (SD) ($n = 18$). Significant differences were determined by Turkey’s test, and any two of the unbracketed letters a, b, c, and d are significantly different. ($P < 0.05$).

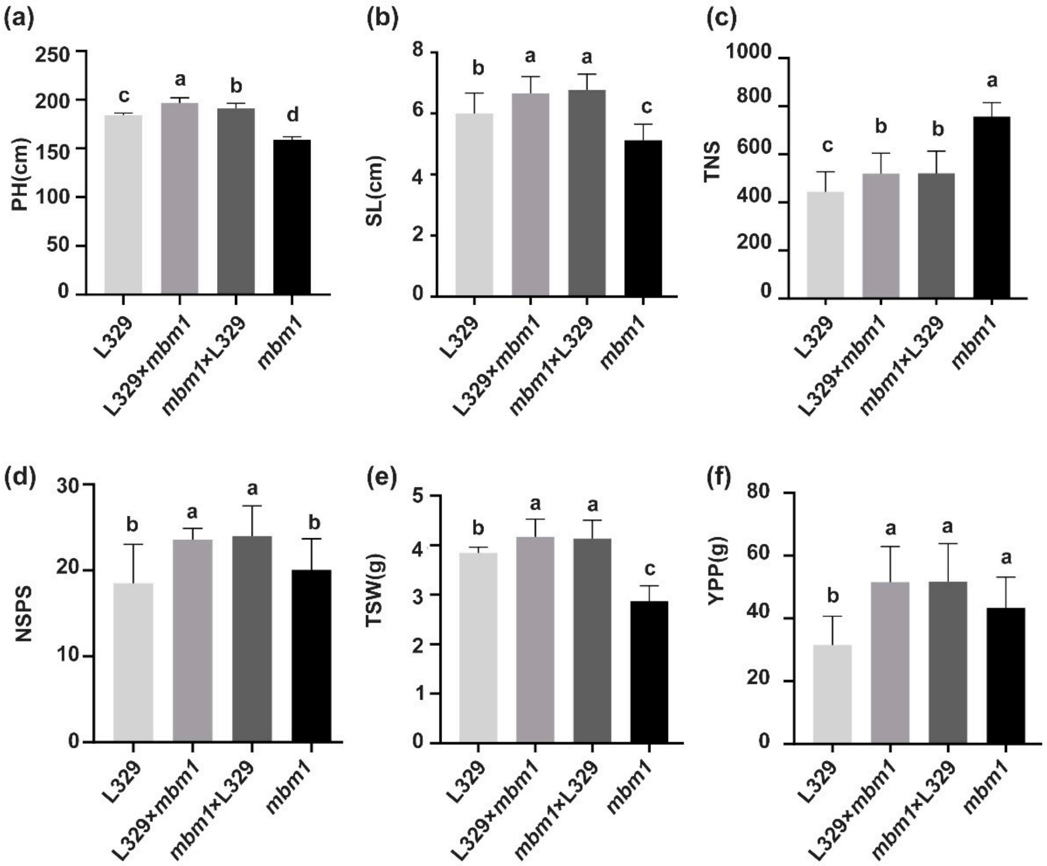

Figure S4. Agronomic traits of reciprocal cross F_1 hybrids between *mbm1* and L329.

(a) Plant height (PH). **(b)** Silique length (SL). **(c)** Total number of siliques (TNS). **(d)** Number of seeds per siliques (NSPS). **(e)** Thousand-seed weight (TSW). **(f)** Yield per plant (YPP). Data are presented as mean ± standard deviation (SD) (n = 18). Significant differences were determined by Turkey's test, and any two of the unbracketed letters a, b, c, and d are significantly different. ($P < 0.05$).

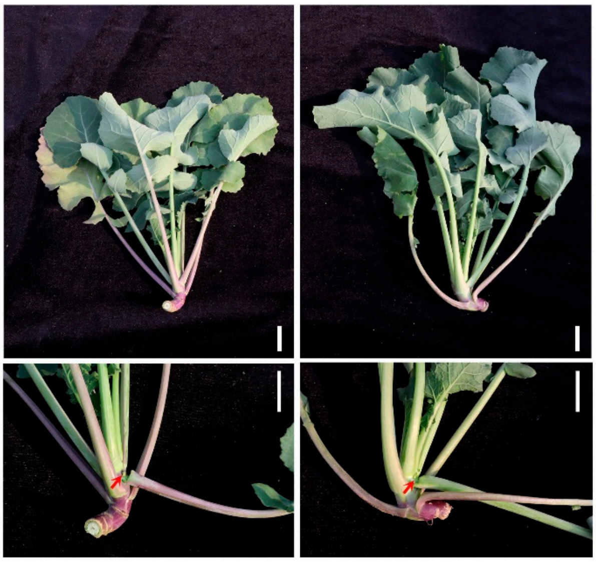

WT ***mbm1***

Figure S5. Plants used for collecting axillary buds for RNA-Seq analysis and sucrose and trehalose content measurement.

The upper panel shows a two-month-old plant after sowing, and the lower panel is a partially enlarged view corresponding to the upper panel. The arrows point to the axillary bud for sampling. Three axillary buds from the base to the upper region were collected from each plant for RNA-seq analysis and sucrose and trehalose content measurement. Scale bar = 3 cm.
